# Presence of Anti-Microbial Antibodies in Liver Cirrhosis – A Tell-Tale Sign of Compromised Immunity?

**DOI:** 10.1371/journal.pone.0012957

**Published:** 2010-09-23

**Authors:** Maria Papp, Gary L. Norman, Zsuzsanna Vitalis, Istvan Tornai, Istvan Altorjay, Ildiko Foldi, Miklos Udvardy, Zakera Shums, Tamas Dinya, Peter Orosz, Bela Lombay, Gabriella Par, Alajos Par, Gabor Veres, Timea Csak, Janos Osztovits, Ferenc Szalay, Peter Laszlo Lakatos

**Affiliations:** 1 2nd Department of Medicine, University of Debrecen, Debrecen, Hungary; 2 INOVA Diagnostics, Inc., San Diego, California, United States of America; 3 Institute of Surgery, University of Debrecen, Debrecen, Hungary; 4 Gastroenterology Department of Medicine, Borsod-Abauj Zemplen County Hospital, Miskolc, Hungary; 5 Department of Medicine, Szent Ferenc Hospital, Miskolc, Hungary; 6 1st Department of Medicine, University of Pecs, Pecs, Hungary; 7 1st Department of Pediatrics, Semmelweis University, Budapest, Hungary; 8 1st Department of Medicine, Semmelweis University, Budapest, Hungary; National University of Singapore, Singapore

## Abstract

**Background:**

Bacterial translocation plays important role in the complications of liver cirrhosis. Antibody formation against various microbial antigens is common in Crohn's disease and considered to be caused by sustained exposure to gut microflora constituents. We hypothesized that anti-microbial antibodies are present in patients with liver cirrhosis and may be associated with the development of bacterial infections.

**Methodology/Principal Findings:**

Sera of 676 patients with various chronic liver diseases (autoimmune diseases:266, viral hepatitis C:124, and liver cirrhosis of different etiology:286) and 100 controls were assayed for antibodies to *Saccharomyces cerevisiae*(ASCA) and to antigens derived from two intestinal bacterial isolates (one gram positive, one gram negative, neither is Escherichia coli). In patients with liver cirrhosis, we also prospectively recorded the development of severe episodes of bacterial infection. ASCA and anti-OMP Plus™ antibodies were present in 38.5% and 62.6% of patients with cirrhosis and in 16% and 20% of controls, respectively (*p*<0.001). Occurrence of these antibodies was more frequent in cases of advanced cirrhosis (according to Child-Pugh and MELD score; *p*<0.001) or in the presence of ascites (*p*<0.001). During the median follow-up of 425 days, 81 patients (28.3%) presented with severe bacterial infections. Anti-microbial antibody titers (*p* = 0.003), as well as multiple seroreactivity (*p* = 0.036), was associated with infectious events. In logistic regression analysis, the presence of ascites (OR:1.62, 95%CI:1.16–2.25), co-morbidities (OR:2.22, 95%CI:1.27–3.86), and ASCA positivity (OR:1.59, 95%CI:1.07–2.36) were independent risk factors for severe infections. A shorter time period until the first infection was associated with the presence of ASCA (*p* = 0.03) and multiple seropositivity (*p* = 0.037) by Kaplan-Meier analysis, and with Child-Pugh stage (*p* = 0.018, OR:1.85) and co-morbidities (*p*<0.001, OR:2.02) by Cox-regression analysis.

**Conclusions/Significance:**

The present study suggests that systemic reactivity to microbial components reflects compromised mucosal immunity in patients with liver cirrhosis, further supporting the possible role of bacterial translocation in the formation of anti-microbial antibodies.

## Introduction

Bacterial infections are associated with increased morbidity and mortality in patients with liver cirrhosis. A wide range of bacterial infections can decompensate liver function and lead to deterioration or death in patients with cirrhosis [1], [2]. Bacterial infection itself, as well as inflammatory mediators, have been confirmed as potential trigger factors in many complications of liver cirrhosis, including variceal bleeding, hepatic encephalopathy, renal failure or impairment in clotting factors [3], [4]. Confirmed risk factors for bacterial infections are advanced disease classified by Child-Pugh stage [5], [6] and the presence of gastrointestinal hemorrhage [7], [8]. Regardless of the severity of the hepatic insufficiency, the development of infection significantly increases the mortality rate. The in-hospital mortality of cirrhotic patients with infection is more than twice that of patients without infection. Various infections are directly responsible for up to 50% of the deaths among patients with liver cirrhosis [1], [6].

An important characteristic of infections in liver cirrhosis is the high incidence of episodes caused by enteric organisms [1], [2]. Bacterial translocation, the passage of bacteria or their products from the gut into the circulation, is a major mechanism in the development of these infections [9]. The remarkable impact of bacterial translocation on the pathogenesis of the disease is highlighted by evidence that selective intestinal decontamination with oral antibiotics reduces the overall risk of infections, and improves short-term survival in high-risk patients [10]. In the absence of overt infections, norfloxacin also normalizes elevated proinflammatory cytokine levels and increases vascular resistance in patients with ascites who present with a high lipopolysaccharide-binding protein (LBP) level [11], [12]. The development of bacterial resistance as a harmful complication of the antibiotic prophylaxis however, is an emerging problem [13], [14]. Detection of bacterial deoxyribonucleic acid (DNA) in blood using the polymerase chain reaction is regarded as a sensitive marker for assessing bacterial translocation [15]. Unfortunately, clinical practice is still lacking reliable and specific serological methods for detecting the presence and extent of bacterial translocation.

Our group has recently hypothesized that the presence of serological responses to various microbial antigens (e.g. phosphopeptidomannan cell-wall component of *Saccharomyces cerevisiae* [ASCA], glycans or complex gut bacterial protein lysates [OMP Plus™]) might be reflections of the sustained systemic exposure to the constituents of the gut microflora due to enhanced bacterial translocation [16]. These anti-microbial antibodies are regarded as characteristic markers for complicated, small-bowel Crohn's disease [17], [18]. Patients with untreated celiac disease however, may also display a similar qualitative and quantitative serological response [16], [19], [20]. Similar to Crohn's disease, the highest antibody prevalence and titers in celiac patients are associated with the most severe form of the clinical presentation: malabsorption. This finding is in line with the hypothesis that among celiac patients, malabsorption is the most pronounced clinical consequence of the intestinal damage [21]. Similarly, in liver cirrhosis inflammation of the small bowel is notable [22] and becomes more pronounced with disease progression. Two thirds of patients with cirrhosis who underwent capsule endoscopy showed mucosal inflammatory-like abnormalities [23]. Alterations of small bowel morphology, like partial villous atrophy and mild-to-moderate increase in lamina propria infiltrate, as well as an increase in intraepithelial lymphocytes were also demonstrated in patients with cirrhosis [24]. Fecal calprotectin levels, which indicate intestinal inflammation, and are widely used to evaluate intestinal inflammation in patients with inflammatory bowel disease were found increased in liver cirrhosis and concentrations were significantly associated with the severity of inflammation [25]. It is thus reasonable to hypothesize that the anti-microbial antibodies may also present in patients with cirrhosis and may be associated with the clinical course of the disease. At present however, there are no data concerning anti-microbial antibodies in liver cirrhosis and its complications.

The aim of our study was to investigate the prevalence of ASCA and anti-OMP Plus™ antibodies in a large Hungarian cohort of patients with chronic liver disease of different etiologies with or without cirrhosis. We also aimed to evaluate the possible interaction between the anti-microbial serologic responses and the disease severity in patients with cirrhosis. Finally, we conducted a follow-up observational study to investigate the presence of these anti-microbial antibodies as markers of bacterial translocation and potential risk factors for the development of severe bacterial infection in liver cirrhosis.

## Methods

### 1. Patient cohort

Six-hundred-seventy-six patients with various chronic liver diseases were investigated. Sera of patients with autoimmune liver diseases (ALD) (n = 266, male/female ratio [m/f]: 102/164, age: 51.1±16.1 years; including primary biliary cirrhosis [PBC, n = 153], primary sclerosing cholangitis [PSC, n = 59], autoimmune hepatitis [AIH, n = 54]), and chronic hepatitis C (chronic HCV, n = 124, m/f: 49/75, age: 53.6±11.7 years) were collected from five Hungarian Hepatology Centers (Debrecen University, Budapest Semmelweis University, Pecs University, Miskolc Borsod-Abauj Zemplen County Hospital, and Miskolc Szent Ferenc Hospital). The diagnosis of primary biliary cirrhosis was based on biochemical evidence of cholestasis, serum anti-mitochondrial antibodies (AMA) and/or PBC-specific AMA-M2 positivity, compatible histology, and the exclusion of extrahepatic homeostasis [26]. The diagnosis of PSC was based on biochemical evidence of cholestasis and the characteristic cholangiographic findings of bile duct stenoses and dilatations. In most cases, it was confirmed by compatible histology findings [27]. The diagnosis of AIH was based on exclusion of other major causes of liver damage, including alcoholic, viral, drug- and toxin-induced, and hereditary liver disease, and using the scoring system of the International AIH Group [28]. The diagnosis of chronic HCV was based on positive HCV ribonucleic acid, elevated liver function tests (>2xULN for more then 6 months) and compatible liver biopsy, if available. The central coordination of sample and database management was done by the Gastroenterology Division of the 2^nd^ Department of Medicine, Debrecen University (M.P. and I.T.). The control group consisted of 100 age- and gender-matched healthy individuals (m/f: 47/53, age: 48.1±15.5 yrs) selected from consecutive blood donors in Debrecen and Budapest. The control subjects did not have any known gastrointestinal or liver diseases.

Serum samples were also obtained from 286 consecutive patients with cirrhosis of different etiologies (m/f: 161/125, age: 56.3±10.7 years) at the Gastroenterology Division of the 2^nd^ Department of Medicine (Debrecen University) during the period from May 2006 to April 2008. The median disease duration was 3 years [IQR, 1–6 years] among cirrhotic patients at the time of the involvement. The clinical data of these patients are summarized in **Table 1**. The etiology of cirrhosis was alcoholic in 180 (63.0%), HCV-related in 87 (30.4%), and various other etiologies in 19 (6.6%). Exclusion criteria were evidence of gastrointestinal bleeding or bacterial infection in the preceding 6 weeks, and prophylactic treatment with non-absorbable antibiotics in the preceding 6 months. The diagnosis of cirrhosis was based on clinical, biochemical, ultrasonographic and, when available, histological features. Clinical data, including age, age at onset, etiology and severity of cirrhosis, presence and grade of ascites, encephalopathy, esophageal varices, prior episode of variceal bleeding, and co-morbidities were collected. Myocardial infarction, congestive heart failure, peripheral arterial disease, cerebrovascular disease, chronic pulmonary disease, chronic renal failure, diabetes mellitus, cancer, including hepatocellular carcinoma, were the co-morbidity diagnoses taken into account during data collection. Severity of the cirrhosis was graded according to the Child–Pugh classification [29] and the model for end-stage liver disease (MELD) score [30] was also calculated. After the blood sampling, cirrhotic patients were followed until April 1, 2009 or death/lost of follow up (median follow-up: 425 days [interquartile range, IQR: 107-732]), for the occurrence of severe bacterial infections. Infectious episodes were identified through inpatient medical records (n = 479), reviewing clinical symptoms, appearance of fever, laboratory data (absolute or relative elevation of the white blood cell count with a left-shift and elevated serum levels of high-sensitivity C-reactive protein and/or procalcitonin), including microbiological culture results, if available, findings of imaging techniques, and the effect of antibiotic treatment by two independent gastroenterologists (M. P. and Zs.V.). Autopsy records (n = 88) were also assessed in cases of death. The following bacterial infections were considered severe: pneumonia (upper respiratory tract infections were excluded), infections of biliary tract (cholecystitis, cholangitis, liver abscess), urinary tract infections (uncomplicated cystitis were excluded), endocarditis, osteomyelitis, infections of skin and soft tissue if associated with bacteraemia, spontaneous bacterial peritonitis and bacteriaemia. The diagnosis of spontaneous bacterial peritonitis was made if the ascitic fluid contained more than 250 polymorphonuclear cells per mm^3^, with or without positive culture, and in the absence of an intra-abdominal source of infection. Bacteriaemia was considered when clinical symptoms and signs of infection were present and confirmed by the microbiological demonstration of the causative organism from the blood culture in the absence of site-specific infection. Other infectious episodes were diagnosed on the basis of conventional criteria.

**Table 1:** Clinical characteristics of patients with liver cirrhosis.

**Liver cirrhosis (n = 286)**	
**Gender (male/female)**	161/125
**Age (mean ± SD, years)**	56.3±10.7
**Median (IQR, years)**	56 (50–64)
**Child-Pugh stage, n**	
**A**	96
**B**	107
**C**	76
**NA**	7
**Meld score (points)**	14.6±6.5
**Serum bilirubin (µmol/L)**	65.8±97.4
**Serum albumin (g/L)**	33.5±7.8
**Platelet (G/L)**	121.1±69.2
**Co-morbidities present, n (%)**	123 (43.1%)
**One**	93
**Two**	24
**Three or more**	6
**HCC, n (%)**	33 (11.5%)
**Mean follow-up ± SD (days)**	440±347
**Median follow-up, IQR (days)**	425 (107–732)
**Patients with severe bacterial infections, n(%)**	81 (28.3%)
**One**	45
**Two**	22
**Three**	14

SD: standard deviation, IQR: inter-quartile range, HCC: hepatocellular carcinoma, NA: not available.

### 2. Ethical considerations

The study protocol was approved by the Ethical and Science Committee of the University of Debrecen. Each patient was informed of the nature of the study and signed an informed consent form.

### 3. Antibody assays

Collected sera were frozen at −80°C until testing. Commercially available ELISA kits were used to test for the presence of ASCA IgG, ASCA IgA, and anti-OMP Plus™ IgA (QUANTA Lite™, INOVA Diagnostics, San Diego, CA) antibodies in sera. Examinations were performed in a blinded fashion, without knowledge of patients' diagnosis or other clinical information in the Laboratory of INOVA Diagnostics, Inc., San Diego, CA by Z.S.

#### ASCA ELISA assay

Both serum IgG and IgA levels of anti-*Saccharomyces cerevisiae* antibodies (ASCA) were evaluated separately according to the manufacturer's protocol (ASCA IgG, ASCA IgA, QUANTA Lite™, INOVA Diagnostics). The results are presented as arbitrary units with a cut-off value for positivity of 25 Units. Sera were documented both, in absolute values and in frequency of positivity.

#### OMP Plus™ ELISA assay

IgA antibodies against multiple bacterial proteins derived from two species of intestinal bacteria (one gram positive and one gram negative) were detected according to the manufacturer's protocol (QUANTA Lite ™ OMP PLUS™ ELISA, INOVA Diagnostics). Neither bacteria are from the phylum proteobacteria, of which *Escherichia* coli is a member. The results are presented as arbitrary units with a cut-off value for positivity of 25 Units. Sera were documented both, in absolute values and in frequency of positivity. For every sample, two analyses on the same plate were carried out.

In order to evaluate the variation in the anti-microbial antibody level, duplicate serum samples were taken from a subgroup of cirrhotic patients (n = 62) at different time points with a median time interval of 204 days [IQR, 71–245 days].

### 4. Detection of lipopolysaccharide-binding protein

Collected sera were frozen at −80°C until testing. LBP was determined in sera of patients with liver cirrhosis and healthy controls by a solid-phase enzyme-linked immunosorbent assay based on the sandwich principle (Hycult Biotechnology, Uden, Netherlands). The lower assay sensitivity limit was 1 ng/mL. For every sample, two analyses on the same plate were carried out and the mean value was used. In a subgroup of cirrhotic patients (n = 102), another serum samples were taken at the time of bacterial infection in order to investigate the changes in serum LBP during bacterial infections. The assays were performed at the Department of Clinical Biochemistry and Molecular Pathology, University of Debrecen.

### 5. Statistical methods

Variables were tested for normality with Shapiro Wilk's W test. T-test with separate variance estimates, χ^2^-test, χ^2^-test with Yates correction, and ANOVA with post hoc Scheffe were used to evaluate differences between cirrhosis and other liver disease controls, as well as within subgroups of patients with cirrhosis, as appropriate. Results are expressed as odds ratio (OR) with 95% confidence intervals (95% CI). Kaplan-Meier survival curves were plotted for analysis with LogRank and Breslow tests. Additionally, logistic regression analysis and forward stepwise Cox-regression analysis was used to assess the association between categorical clinical variables and risk of and time to significant clinical infection. A *p* value of <0.05 was considered as significant. For statistical analysis, SPSS15.0 (SPSS Inc, Chicago, IL) was used, with the help of a statistician (Dr. Peter Vargha).

## Results

### 1. Anti-microbial serological markers in patients with chronic liver diseases

The prevalence rates of ASCA and anti-OMP Plus™ antibodies in patients with various chronic liver diseases are presented in **Table 2**. The rate of ASCA IgA and/or IgG and also anti-OMP Plus™ IgA seropositivity was greatly elevated in patients with liver cirrhosis compared to healthy controls (OR_ASCAeither_: 3.28, 95% CI: 1.83–5.89; OR_anti-OMP Plus_™: 6.69, 95% CI: 3.88–11.55) or chronic liver diseases without cirrhosis (*p*<0.001 for both). Seropositivity rates were even lower in patients with chronic HCV without cirrhosis as compared to the controls, other liver diseases, or patients with cirrhosis (*p*<0.001 for all). The rates of ASCA and anti-OMP Plus™ positivity were not overall different overall in patients with autoimmune liver disease without cirrhosis as compared to the controls. A more subtle analysis of this group revealed that the frequency of ASCA positivity was also significantly higher in a small group of patients with PSC (*p* = 0.04), but not in those with PBC or AIH. Regarding anti-OMP Plus™ IgA, no similar tendency was observed. In patients with cirrhosis, the serological profile was not different based on disease etiology (data not shown). 10.4% of patients with cirrhosis were triple-positive for ASCA IgA, ASCA IgG, and anti-OMP Plus™ IgA antibodies, compared with only 1% of control subjects (*p<*0.0001). Only one patient was triple-positive in the ALD group and none in the chronic HCV group.

**Table 2:** Anti-microbial serological markers in patients with chronic liver diseases and healthy controls.

		ASCA	Anti-OMP Plus™
	Number	IgA and/or IgG	IgA
**Liver cirrhosis with various etiology**	286	110 (38.5%)*	179 (62.6%)*
**Chronic HCV without cirrhosis**	124	11 (8.9%)#	3 (2.4%)#
**Autoimmune liver diseases without cirrhosis**	266	59 (22.2%)	44 (16.5%)
**PBC**	153	31 (20.3%)	26 (17.0%)
**PSC**	59	18 (30.5%)&,φ	9 (15.3%)⊥
**AIH**	54	10 (18.5%)	9 (16.7%)
**Healthy controls**	100	16 (16%)	20 (20%)

AIH = autoimmune hepatitis, HCV = viral hepatitis C, PBC = primary biliary cirrhosis, PSC = primary sclerosing cholangitis.

*p<0.001 between liver cirrhosis and chronic HCV, autoimmune liver diseases, healthy controls.

# *p*<0.001 between chronic HCV patients and autoimmune liver diseases, healthy controls.

& *p* = 0.04 between PSC and healthy controls.

φ *p*<0.001 between PSC and chronic HCV.

⊥ *p*<0.01 between PSC and chronic HCV.

by using Fisher's exact test or χ^2^-test with Yates correction if appropriate.

**Figure 1:**
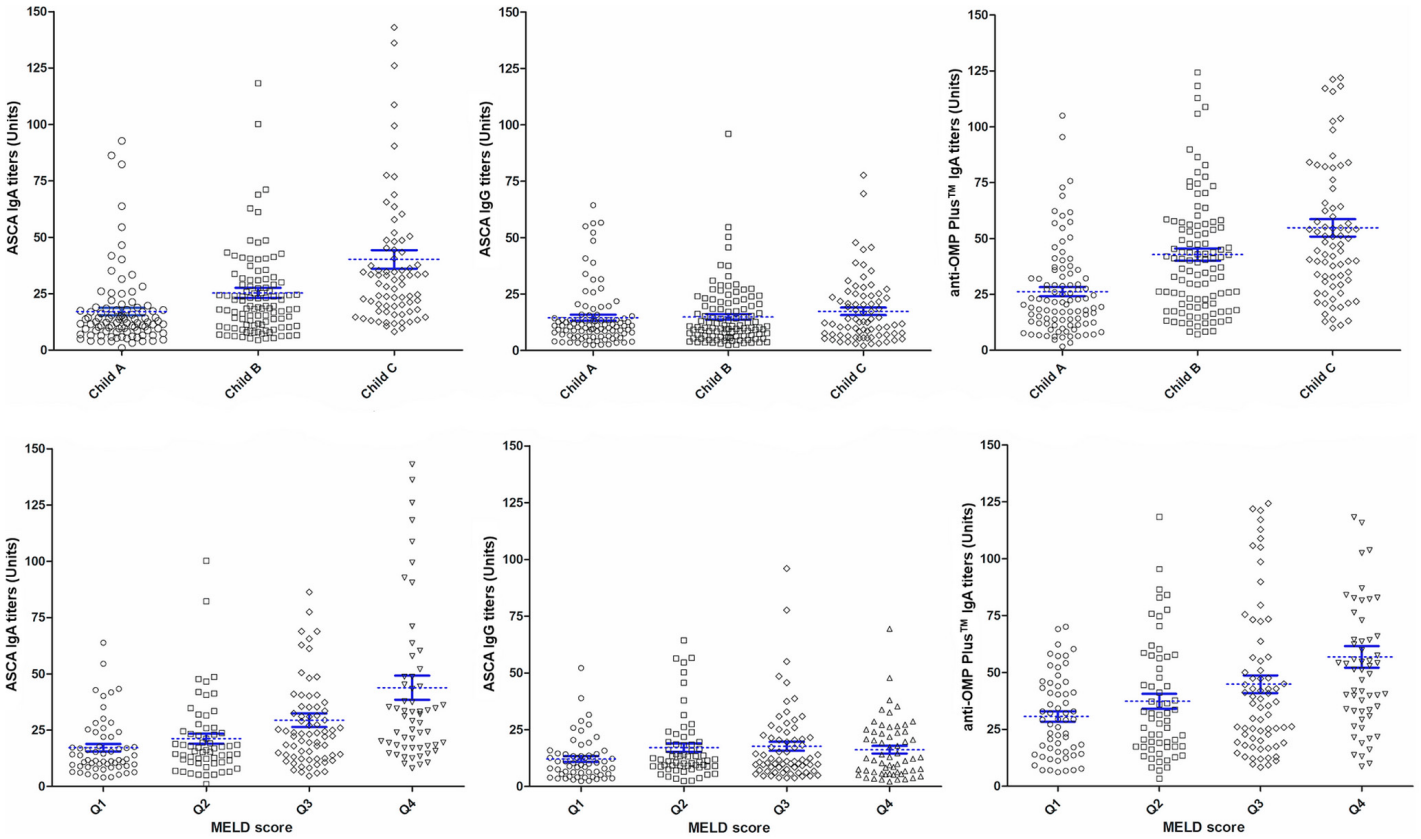
Anti-microbial antibody levels in patients with cirrhosis with various levels of severity, as depicted either by Child-Pugh stages (A) or MELD score (B). **A**. Individual values are shown by black spots. Mean values with standard error bars are indicated in blue. Cut-off values for positivity are 25 Units for all antibodies. *P*<0.001 between all groups by ANOVA post hoc Scheffe for ASCA IgA and anti-OMP Plus™ IgA *P = NS* for ASCA IgG. **B**. MELD Q1-Q4 represent the groups of patients broken down by quartile: quartile1 patients have the lowest severity up to quartile4, representing patients with the highest level of severity. *P*<0.001 between all groups by ANOVA post hoc Scheffe for ASCA IgA and anti-OMP Plus™ IgA *P = NS* for ASCA IgG.

In patients with liver cirrhosis, seropositivity rates increased gradually according to disease severity, as rated by the Child-Pugh stage (see **Table 3**) or MELD score (data not shown), for both ASCA IgA and anti-OMP Plus™ IgA. 18.4% of patients with Child C cirrhosis were triple-positive for ASCA IgA, ASCA IgG, and anti-OMP Plus™ IgA antibodies, compared with only 4.2% in Child A and 10.3% in Child B groups (*p*<0.01 for both). The same association was found if the triple seropositivity rate was calculated for the MELD inter-quartile ranges (IQR) (1^st^ quartile: 1.7% 2^nd^ quartile: 6.6% 3^rd^ quartile: 14.3% 4^th^ quartile: 20.3%, *p*<0.01). Similarly to the rates of seropositivity, the more severe the disease, the higher were the titers of the ASCA IgA and anti-OMP Plus™ IgA, but not the titer of ASCA IgG (see **Figure 1**).

**Table 3:** Anti-microbial serological markers in patients with liver cirrhosis according to the disease severity depicted by Child-Pugh stages.

	Child-Pugh stages		
	A (n = 96)	B (n = 107)	C (n = 76)
**ASCA IgA and/or IgG**	23 (24.0%)	42 (39.3%)	45 (59.2%)*
ASCA IgA	15 (15.6%)	37 (34.6%)	43 (56.6%)*
ASCA IgG	14 (14.6%)	16 (15.0%)	18 (23.7%)
**Anti-OMP Plus™ IgA**	38 (39.6%)	77 (72.0%)	64 (84.2%)*
**Multiple seropositivity**	18 (17.7%)	38 (35.5%)	44 (57.9%)*

*P<0.001 for both by linear-by-linear association.

Child-Pugh stage were not available for 7 patients.

The occurrence of either individual anti-microbial antibodies or multiple seropositivity was associated with the presence of ascites (OR_ASCA either_: 1.93, 95% CI: 1.19–3.14; OR_anti-OMP Plus_™: 3.08, 95% CI: 1.86–5.11) (see **Table 4**) as well as the quantitative anti-OMP Plus™ IgA serological response (median, no ascites vs. ascites: 25.4 vs. 41.5 Units, *p<*0.001).

**Table 4:** Anti-microbial serological markers in cirrhotic patients according to the absence or presence of ascites.

	Patients without ascites	Patients with ascites
	(n = 150)	(n = 136)
**ASCA IgA and/or IgG**	46 (30.7%)	63 (46.3%)*
ASCA IgA	39 (26.0%)	58 (42.6%)*
ASCA IgG	25 (16.7%)	24 (17.6%)
**Anti-OMP Plus™ IgA**	75 (50.0%)	103 (75.7%)#
**Multiple seropositivity**	39 (26.0%)	60 (44.1%)*

*p<0.01,

# *p*<0.001 between cirrhotic patients with or without ascites by χ^2^-test with Yates correction and linear-by-linear association for serological responses.

Median serum LBP levels were not statistically different between patients with liver cirrhosis and healthy subjects (21, 860 vs. 19, 333 ng/ml, p = 0.08). No correlation was found between LBP levels and the disease severity or the presence of ascites. Furthermore, serum LBP levels were not different in cirrhotic patients with and without ASCA IgG/IgA, and anti-OMP IgA antibodies or if a combination of anti-microbial antibodies was used (data not shown).

### 2. Clinical and laboratory predictors for the development of severe infection

#### Bacterial infections: general characteristics and microbiologic results

Among the 286 cirrhotic patients, a total of 176 severe infectious episodes were identified during hospitalizations. 28.3% of the patients presented with some type of infection (**Table 1**). Of the patients with infection, 44.4% suffered more than one episode. The distribution of different severe infections was as follows: 28.2% spontaneous bacterial peritonitis, 21.5% pneumonia, 12.5% urinary tract infection, 8.7% skin and soft tissue infections and 12.6% miscellaneous. The origin of the infection could not be identified in 16.5% of the cases. Bacteria were gram negative in 57.1% and gram positive in 42.9% of positive cases. There was no difference in the proportion of the different types of bacterial infection according to the anti-microbial serology status (data not shown).

#### Clinical and laboratory predictors for the development of bacterial infection

Of the clinical factors, the disease severity by Child-Pugh stage (*p* = 0.035), the presence of ascites (OR: 2.83; 95% CI: 1.64–4.86, *p*<0.001) and co-morbidities (OR: 2.42; 95% CI: 1.47–4.24, *p* = 0.001) were found to be risk factors for the development of severe infections, using univariate analysis (χ2-test or χ2-test with Yates correction).

Considering the anti-microbial antibodies individually, both the presence (OR: 1.73, 95% CI: 1.02–2.92, p = 0.041) and the type of ASCA positivity (not positive: 24.4%, IgA- or IgG- positive: 30.8%, IgA- and IgG-positive: 43.8%, *p* = 0.015 by linear-by-linear association) were associated with the development of severe infections. The same tendency was also observed for anti-OMP Plus™ IgA positivity (21.5% vs. 31.5%, *p* = 0.069), but not for LBP. Similarly, the severe infection rate increased in parallel to the extent of both ASCA IgG and IgA serological responses (IgA IQR titers: 1^st^ quartile: 16.9%, 2^nd^ quartile: 28.8%, 3^rd^ quartile: 30.3% and 4^th^ quartile: 36.8%; ASCA IgG: 1^st^ quartile: 18.6%, 2^nd^ quartile: 24.7%, 3^rd^ quartile: 34.7% and 4^th^ quartile: 35.2%; *p = *0.003 by linear by linear association for both).

The presence of serological response to multiple markers was also significantly associated with the probability of developing severe infections. The rate of the severe infection was 21.1%, 27.7%, 31.4% and 43.3% based on seropositivity, positive for 0, 1, 2 and 3 markers, respectively (*p* = 0.036 by linear-by-linear association).

In a logistic regression analysis, the presence of ascites, co-morbidities and ASCA positivity, but not LBP were independent predictors of development of severe infections (**Table 5**).

**Table 5:** Logistic regression: Predictive factors for severe bacterial infection in patients with liver cirrhosis.

Factor	Coefficient	*P* value	OR	95% CI
**Female gender**	0.355	0.228	1.43	0.80–2.54
**Child-Pugh stage**	−0.096	0.669	0.91	0.58–1.41
**Ascites**	0.481	0.004	1.62	1.16–2.25
**LBP level (>26, 236 ng/ml)**	−0.094	0.85	0.92	0.44–1.89
**Co-morbidities**	0.796	0.005	2.22	1.27–3.86
**ASCA IgA and/or IgG**	0.491	0.018	1.63	1.09–2.45

The coefficient is equivalent to the natural log of the OR; *p* value: level of significance;

OR: odds ratio; 95% CI: 95% confidence interval.

### 3. Clinical and laboratory parameters associated with time to first severe infection

**Figure 2:**
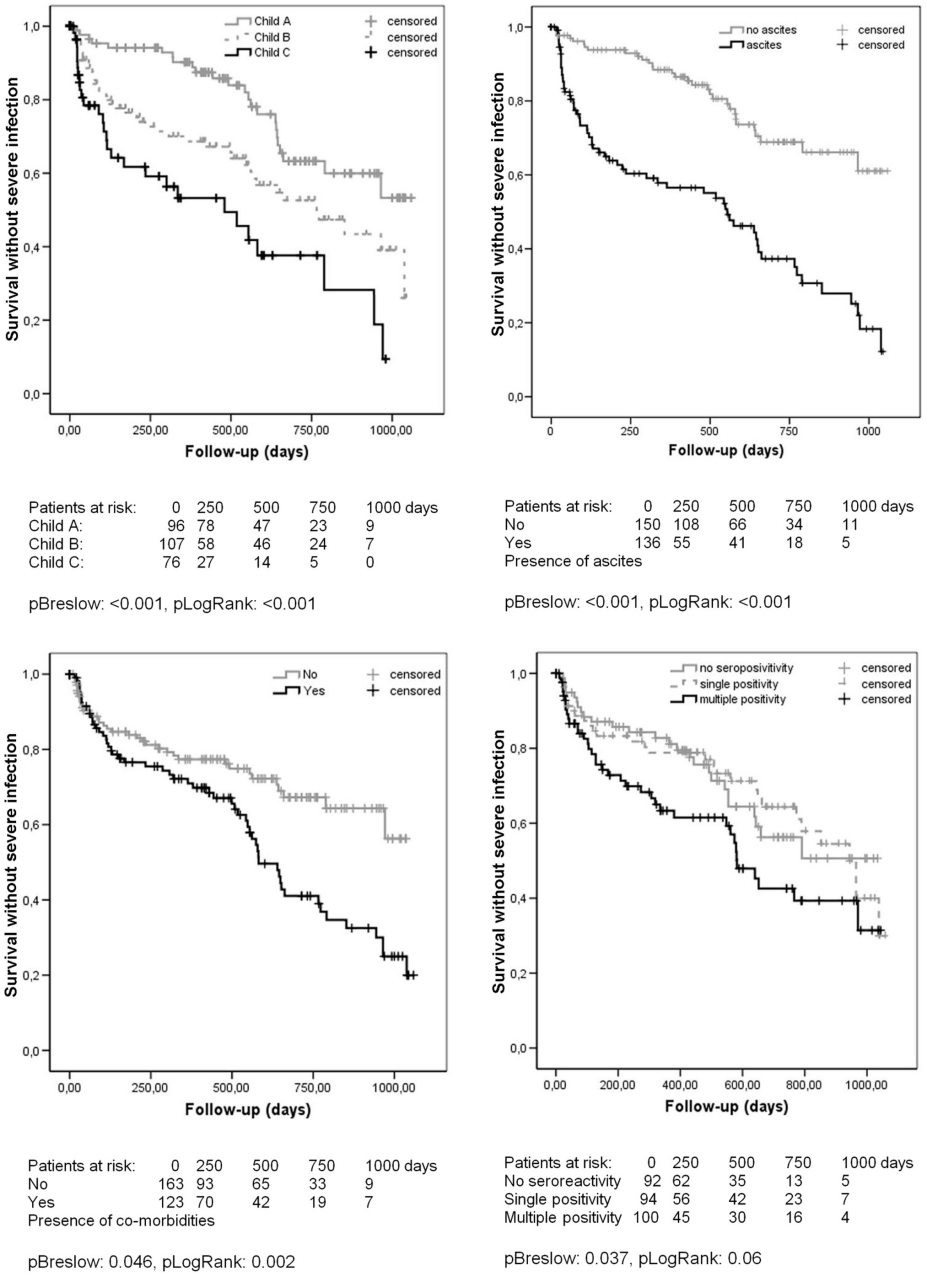
Association between Child-Pugh stages (A), presence of ascites (B), co-morbidities (C), seropositivity to ASCA/anti-OMP Plus™ (D) and the development of severe bacterial infection in patients with liver cirrhosis. Infection-free survival refers to the proportion of patients in the cohort without infection at a given time during the follow-up. A. Patients with Child C stage cirrhosis were at higher risk for developing severe bacterial infections compared to patients with Child A or B disease. B. Patients with ascites were at higher risk for developing severe bacterial infections compared to patients without ascites. C. Patients with co-morbidity were at higher risk for developing severe bacterial infections compared to patients without co-morbidity. D. Patients with multiple seropositivity were at higher risk for developing severe bacterial infections compared to seronegative ones.

In a Kaplan-Meier analysis, Child-Pugh stages, presence of ascites, and co-morbidities were associated with time to first severe infection by Breslow and LogRank (**Figure 2**). Of the serologic markers, presence of ASCA (pBreslow = 0.03) and seropositivity to multiple markers (pBreslow: 0.037) were associated with time to first severe infection (**Figure 2**).

In a Cox-regression analysis, the Child-Pugh stage (*p*<0.001) and co-morbidities (*p* = 0.006, OR: 1.83), but not seroreactivity were associated with shorter time until the first infection (**Table 6**).

**Table 6:** Summary of Cox model: factors affecting time to first severe bacterial infection.

	*p*	Hazard Ratio	95% CI
**Co-morbidities**	0.006	1.83	1.19–2.80
**Child-Pugh stage**	<0.001		
**Child A**	reference		
**Child B**	0.048	1.71	1.01–2.91
**Child C**	<0.001	3.26	1.90–5.59
**Seropositivity to ASCA/anti-OMP Plus™**	0.77	1.04	0.78–1.39

*p* value: level of significance; 95% CI: 95% confidence interval.

### 4. Clinical and laboratory parameters associated with mortality

In total, 88 patients (30.8%) died. Of the clinical factors, the disease severity by Child-Pugh stage (*p*<0.001), the presence of ascites (OR: 3.17; 95% CI: 1.87–5.38, *p*<0.001) and co-morbidities (OR: 1.71; 95% CI: 1.03–2.83, *p* = 0.038) were found to be risk factors for mortality, using univariate analysis (χ2-test or χ2-test with Yates correction) similar to the presence of both ASCA IgA and IgG (OR: 1.75; 95% CI: 1.05–2.91, *p* = 0.032), anti-OMP Plus™ (OR: 2.08; 95% CI: 1.20–3.62, *p* = 0.009) and multiple serology positivity (p = 0.037). In addition, the presence of ASCA IgA and/or IgG (pLogRank = 0.045, pBreslow = 0.05), anti-OMP Plus (pLogRank = 0.009, pBreslow = 0.02) or multiple seropositivity (pLogRank = 0.01, pBreslow = 0.02) were also associated with time to death in a Kaplan-Meier analysis.

In contrast by logistic regression analysis, only Child-Phugh stage (p<0.001) and presence of co-morbidities (p = 0.028–0.30), and by Cox-regression analysis only Child-Pugh stage (p<0.001) was associated with short term mortality and time to death.

## Discussion

To our knowledge, this is the first report to investigate the complex associations between anti-microbial serological responses and the disease etiology along with severity in a large cohort of patients suffering from various chronic liver diseases. In the present study, we demonstrated that the presence of anti-microbial antibodies in chronic liver diseases was mainly associated with the presence of cirrhosis and was unrelated to the etiology. In the absence of liver cirrhosis, the presence of antibodies was similar to that observed in the controls. The rate of ASCA positivity in patients with ALD and chronic HCV was equal to the findings of previous studies [31], [32], [33]. As a result of the lower antibody rate in their healthy control group, however, a significantly higher ASCA prevalence was found in the ALD compared to healthy controls.

The exact mechanism behind anti-microbial antibody formation is not fully understood. In the present study we aimed to provide new pieces of this puzzle. The presence of anti-microbial antibodies is not specific for certain diseases but the mechanism behind their formation might be specific, however, linking primarily different diseases. We hypothesized that the development of anti-microbial antibodies is triggered by sustained bacterial translocation from the gut to the systemic circulation as a consequence of the enhanced intestinal permeability and compromised mucosal immunity.

The OMP Plus™ IgA assay detects antibodies to a complex mixture of bacterial proteins derived from one species of gram positive and one species of gram negative gut bacteria having a potential to translocation from the gut to the systemic circulation. ASCAs are directed primarily against mannan cell-wall components of different microorganisms [34] and occurred frequently in some diseases associated with chronic bacterial translocation (e.g. Crohn's disease, celiac disease) [16], [17], [18], [19], [20]. Bacterial translocation is frequently reported in patients with liver cirrhosis, especially in those with more severe liver dysfunction [35], as well as in experimental cirrhosis in the presence of ascites [36]. The present study demonstrated that both the seropositivity rates and antibody titers showed a gradual increase according to the severity of liver cirrhosis- Anti-microbial antibody titers reached the highest value in patients with advanced liver cirrhosis. Furthermore, both the qualitative and quantitative serological responses were also associated with the presence of ascites. The mechanisms involved in bacterial translocation are related to the progression of the liver disease [37], [38]. Aerobic facultative gram negative bacilli, which are only present in low numbers in the small bowel of normal subjects, are found to be increased in jejunal flora of many patients with cirrhosis. Small intestinal bacterial overgrowth exists in approximately 60% of patients with cirrhosis [39], particularly in those with ascites and advanced liver dysfunction [40]. Impaired small intestinal motility [41] and decreased immunological defense are thought to play a major role in its development [9]. Disruption of the gut-barrier integrity secondary to mucosal blood flow changes, oxidative damage, upregulation of proinflammatory cytokines and nitric oxide also enhance bacterial translocation [42]. In the present study, the dominant elevation of IgA type antibodies over IgG one may suggest that compromised mucosal immunity play a principal role in the development of anti-microbial antibody formation in patients with advanced liver cirrhosis.

Gram negative bacteria are the most frequently translocating bacteria in patients with liver cirrhosis [9]. Anti-gal antibodies directed to the surface antigen of gram negative bacteria have been found to be significantly associated with individuals with stage III or greater fibrosis, regardless of the etiology of liver diseases. Patients with increased levels of anti-gal antibody had increased levels of endotoxin and other markers of bacterial exposure [43]. In our study, anti-OMP Plus™ IgA antibodies, which include antibodies to targeting bacterial proteins derived from both gram negative and gram positive gut bacteria, were detected in 62.6% of antibodies in cirrhotic patients, about 60% more frequent than ASCA IgG and/or IgA antibodies.

Furthermore, PSC was the only subgroup in which we detected significantly higher prevalence of ASCA, even in the lack of advanced liver cirrhosis. ASCA positivity was comparable to that in the study of *Muratori et al.* (30.5% vs. 44%) [31] and did not differ according to the concomitant inflammatory bowel disease. This finding also supports our hypothesis that anti-microbial antibody formation is particularly driven by sustained bacterial translocation. Because of the development of biliary stenotic lesions in PSC, the likelihood of infection is relatively high. These lesions have been correlated with increased susceptibility to infections by enteric gram negative pathogens [44]. Increased prevalence of anti-microbial antibodies in other clinical conditions complicated by bacterial translocation would also support our hypothesis. However, no data are available in the current literature regarding this.

Genetically-based loss of tolerance toward the gut flora was suggested to trigger the formation of anti-microbial antibodies in Crohn's disease [45]. Enhanced bacterial translocation associated to nucleotide-binding oligomerization containing domain 2/caspase recruitment domain 15 (NOD2/CARD15) variants only explains partially high prevalence of these antibodies. The occurrence of ASCA was higher by 25% in Crohn's disease patients carrying at least one NOD2/CARD15 mutation as compared to the non-carrier ones [46], [47], [48]; however, the ASCA prevalence was also high in this latter group. Furthermore, the fact that there is a considerably higher prevalence of anti-microbial antibodies both in liver cirrhosis and untreated celiac disease without higher carriage of variant NOD2/CARD15 alleles [16], [49], [50], suggests that genetically-based loss of tolerance toward the gut flora [45] is not the only mechanism triggering the anti-microbial antibody formation. The acquired structural and functional impairment of the small bowel as well as the variation in the microbial flora may be an important, if not primary, factor inducing bacterial translocation with an enhanced anti-microbial antibody formation in all of these gastrointestinal disorders.

Elevated LBP level was suggested to be related to bacterial passage from the gut to the circulation without overt infection in cirrhotic patients with ascites on the basis of the fact that patients with increased serum LBP level were four times more likely to have severe bacterial infection during follow-up than patients with normal LBP [51]. For this reason, we examined whether any association existed between serum LBP level and the presence of anti-microbial antibodies. Serum LBP levels were not different in patients with and without ASCA IgG/IgA, and anti-OMP Plus™ IgA antibodies or in the case a combination of these antibodies were used. At the same time, LBP was found to be an acute phase marker during bacterial infection. The best accuracy for LBP was detected at 26 236 ng/L based on the ROC analysis with a sensitivity of 60.2% and a specificity of 75.1%.

One of the limitations of our study is that the expression of the anti-microbial antibodies was not assessed directly in the intestinal mucosa or in the organs to which gut bacteria are translocated (e.g. liver or ascites). Instead, to support our hypothesis that anti-microbial antibody formation might be a consequence of chronic bacterial translocation, we conducted a follow-up observational study to examine whether the presence of these antibodies may have been associated to the development of severe bacterial infections. The presence and the extent of anti-microbial serological response was found to be associated to severe bacterial infections overall and the time to first infection was also shorter in patients with multiple seropositivity. Recently common variants of nucleotide-binding oligomerization containing domain 2/caspase recruitment domain 15 (NOD2/CARD15) linked to impaired mucosal barrier function were also reported to be risk factors for spontaneous bacterial peritonitis in liver cirrhosis [52]. On the contrary to genetic variation of this and other pathogen-associated molecular patterns' receptors [53], the presence of anti-microbial antibodies were not associated with either the infection-related or the overall mortality in multivariate analysis suggesting that they have no role in the progression (injury, fibrogenesis) and the bacterial complication of the liver cirrhosis. Similarly, no data suggest any pathogenetic role in other diseases associated with high frequency of anti-microbial antibodies for the present. However, anti-microbial antibodies might be feasible medium-term biomarkers for future clinical applications regarding severity of liver cirrhosis and prediction of bacterial complications. Concuring with the findings of *Rieder et al.* [54] in patients with inflammatory bowel disease, we found the ASCA and anti-OMP™antibody stability to be acceptable on the medium-term. Duplicate samples, taken from the same patients at different time points and assayed for these antibodies, showed no significant variation in serum levels. Only 9.7% (6/62) of the patients changed their antibody status during the median period of 204 days [IQR, 71–245 days].

In summary, the presence of anti-microbial antibodies was common in patients with liver cirrhosis and was associated with advanced disease, the existence of portal hypertension, but not with the disease etiology. Serological response to various microbial components might be considered as a universal marker for the enhanced translocation of gut microflora or a reflection of compromised mucosa immunity. To determine whether assessment of the anti-microbial serological markers has any additional benefit over the currently used factors (disease severity stage, presence of ascites) for the prediction of infections in patients with cirrhosis further long-term prospective studies are warranted.

## References

[1] Borzio M, Salerno F, Piantoni L, Cazzaniga M, Angeli P (2001). Bacterial infection in patients with advanced cirrhosis: a multicentre prospective study.. Dig Liver Dis.

[2] Tandon P, Garcia-Tsao G (2008). Bacterial infections, sepsis, and multiorgan failure in cirrhosis.. Semin Liver Dis.

[3] Thalheimer U, Triantos CK, Samonakis DN, Patch D, Burroughs AK (2005). Infection, coagulation, and variceal bleeding in cirrhosis.. Gut.

[4] Garcia-Tsao G, Wiest R (2004). Gut microflora in the pathogenesis of the complications of cirrhosis.. Best Pract Res Clin Gastroenterol.

[5] Yoshida H, Hamada T, Inuzuka S, Ueno T, Sata M (1993). Bacterial infection in cirrhosis, with and without hepatocellular carcinoma.. Am J Gastroenterol.

[6] Caly WR, Strauss E (1993). A prospective study of bacterial infections in patients with cirrhosis.. J Hepatol.

[7] Bernard B, Grangé JD, Khac EN, Amiot X, Opolon P (1999). Antibiotic prophylaxis for the prevention of bacterial infections in cirrhotic patients with gastrointestinal bleeding: a meta-analysis.. Hepatology.

[8] Soares-Weiser K, Brezis M, Tur-Kaspa R, Leibovici L (2002). Antibiotic prophylaxis for cirrhotic patients with gastrointestinal bleeding.. Cochrane Database Syst Rev.

[9] Wiest R, Garcia-Tsao G (2005). Bacterial translocation (BT) in cirrhosis.. Hepatology.

[10] Saab S, Hernandez JC, Chi AC, Tong MJ (2009). Oral antibiotic prophylaxis reduces spontaneous bacterial peritonitis occurrence and improves short-term survival in cirrhosis: a meta-analysis.. Am J Gastroenterol.

[11] Albillos A, de la Hera A, González M, Moya JL, Calleja JL (2003). Increased lipopolysaccharide binding protein in cirrhotic patients with marked immune and hemodynamic derangement.. Hepatology.

[12] Albillos A, Hera Ad Ade L, Reyes E, Monserrat J, Muñoz L (2004). Tumour necrosis factor-alpha expression by activated monocytes and altered T-cell homeostasis in ascitic alcoholic cirrhosis: amelioration with norfloxacin.. J Hepatol.

[13] Singh N, Wagener MM, Gayowski T (2003). Changing epidemiology and predictors of mortality in patients with spontaneous bacterial peritonitis at a liver transplant unit.. Clin Microbiol Infect.

[14] Fernández J, Navasa M, Planas R, Montoliu S, Monfort D (2007). Primary prophylaxis of spontaneous bacterial peritonitis delays hepatorenal syndrome and improves survival in cirrhosis.. Gastroenterology.

[15] Zapater P, Francés R, González-Navajas JM, de la Hoz MA, Moreu R (2008). Serum and ascitic fluid bacterial DNA: a new independent prognostic factor in noninfected patients with cirrhosis.. Hepatology.

[16] Papp M, Foldi I, Altorjay I, Palyu E, Udvardy M (2009). Anti-microbial antibodies in celiac disease: trick or treat?. World J Gastroenterol.

[17] Mow WS, Vasiliauskas EA, Lin YC, Fleshner PR, Papadakis KA (2004). Association of antibody responses to microbial antigens and complications of small bowel Crohn's disease.. Gastroenterology.

[18] Papp M, Altorjay I, Norman GL, Shums Z, Palatka K (2007). Seroreactivity to microbial components in Crohn's disease is associated with ileal involvement, noninflammatory disease behavior and NOD2/CARD15 genotype, but not with risk for surgery in a Hungarian cohort of IBD patients.. Inflamm Bowel Dis.

[19] Toumi D, Mankai A, Belhadj R, Ghedira-Besbes L, Jeddi M (2007). Anti-Saccharomyces cerevisiae antibodies in coeliac disease.. Scand J Gastroenterol.

[20] Granito A, Muratori L, Muratori P, Guidi M, Lenzi M (2006). Anti-saccharomyces cerevisiae antibodies (ASCA) in coeliac disease.. Gut.

[21] Hill PG, Holmes GK (2008). Coeliac disease: a biopsy is not always necessary for diagnosis.. Aliment Pharmacol Ther.

[22] Saitoh O, Sugi K, Lojima K, Matsumoto H, Nakagawa K (1999). Increased prevalence of intestinal inflammation in patients with liver cirrhosis.. World J Gastroenterol.

[23] De Palma GD, Rega M, Masone S, Persico F, Siciliano S (2005). Mucosal abnormalities of the small bowel in patients with cirrhosis and portal hypertension: a capsule endoscopy study.. Gastrointest Endosc.

[24] Bhonchal S, Nain CK, Prasad KK, Nada R, Sharma AK (2008). Functional and morphological alterations in small intestine mucosa of chronic alcoholics.. J Gastroenterol Hepatol.

[25] Yagmur E, Schnyder B, Scholten D, Schirin-Sokhan R, Koch A (2006). [Elevated concentrations of fecal calprotectin in patients with liver cirrhosis].. Dtsch Med Wochenschr.

[26] Kaplan MM, Gershwin ME (2005). Primary biliary cirrhosis.. N Engl J Med.

[27] Weismüller TJ, Wedemeyer J, Kubicka S, Strassburg CP, Manns MP (2008). The challenges in primary sclerosing cholangitis–aetiopathogenesis, autoimmunity, management and malignancy.. J Hepatol.

[28] Alvarez F, Berg PA, Bianchi FB, Bianchi L, Burroughs AK (1999). International Autoimmune Hepatitis Group Report: review of criteria for diagnosis of autoimmune hepatitis.. J Hepatol.

[29] Durand F, Valla D (2005). Assessment of the prognosis of cirrhosis: Child-Pugh versus MELD.. J Hepatol.

[30] Kamath PS, Kim WR (2007). The model for end-stage liver disease (MELD).. Hepatology.

[31] Muratori P, Muratori L, Guidi M, Maccariello S, Pappas G (2003). Anti-Saccharomyces cerevisiae antibodies (ASCA) and autoimmune liver diseases.. Clin Exp Immunol.

[32] Reddy KR, Colombel JF, Poulain D, Krawitt EL (2001). Anti-Saccharomyces cerevisiae antibodies in autoimmune liver disease.. Am J Gastroenterol.

[33] Sakly W, Jeddi M, Ghedira I (2008). Anti-Saccharomyces cerevisiae antibodies in primary biliary cirrhosis.. Dig Dis Sci.

[34] Papp M, Norman GL, Altorjay I, Lakatos PL (2007). Utility of serological markers in inflammatory bowel diseases: gadget or magic?. World J Gastroenterol.

[35] Cirera I, Bauer TM, Navasa M, Vila J, Grande L (2001). Bacterial translocation of enteric organisms in patients with cirrhosis.. J Hepatol.

[36] Garcia-Tsao G, Lee FY, Barden GE, Cartun R, West AB (1995). Bacterial translocation to mesenteric lymph nodes is increased in cirrhotic rats with ascites.. Gastroenterology.

[37] Guarner C, Soriano G (2005). Bacterial translocation and its consequences in patients with cirrhosis.. Eur J Gastroenterol Hepatol.

[38] Norman K, Pirlich M (2008). Gastrointestinal tract in liver disease: which organ is sick?. Curr Opin Clin Nutr Metab Care.

[39] Bauer TM, Steinbrückner B, Brinkmann FE, Ditzen AK, Schwacha H (2001). Small intestinal bacterial overgrowth in patients with cirrhosis: prevalence and relation with spontaneous bacterial peritonitis.. Am J Gastroenterol.

[40] Casafont Morencos F, de las Heras Castaño G, Martín Ramos L, López Arias MJ, Ledesma F (1996). Small bowel bacterial overgrowth in patients with alcoholic cirrhosis.. Dig Dis Sci.

[41] Gunnarsdottir SA, Sadik R, Shev S, Simrén M, Sjövall H (2003). Small intestinal motility disturbances and bacterial overgrowth in patients with liver cirrhosis and portal hypertension.. Am J Gastroenterol.

[42] Balzan S, de Almeida Quadros C, de Cleva R, Zilberstein B, Cecconello I (2007). Bacterial translocation: overview of mechanisms and clinical impact.. J Gastroenterol Hepatol.

[43] Mehta A, Loarca L, Long RE, Comunale M, Block TM (2009). Discovery of an antibody in patients with liver disease that promotes bacterial growth and is associated with markers of endtoxin exposure.. AASLD MON-A.

[44] Pohl J, Ring A, Stremmel W, Stiehl A (2006). The role of dominant stenoses in bacterial infections of bile ducts in primary sclerosing cholangitis.. Eur J Gastroenterol Hepatol.

[45] Vermeire S, Vermeulen N, Van Assche G, Bossuyt X, Rutgeerts P (2008). (Auto)antibodies in inflammatory bowel diseases.. Gastroenterol Clin North Am.

[46] Henckaerts L, Pierik M, Joossens M, Ferrante M, Rutgeerts P (2007). Mutations in pattern recognition receptor genes modulate seroreactivity to microbial antigens in patients with inflammatory bowel disease.. Gut.

[47] Mei L, Targan SR, Landers CJ, Dutridge D, Ippoliti A (2006). Familial expression of anti-Escherichia coli outer membrane porin C in relatives of patients with Crohn's disease.. Gastroenterology.

[48] Papp M, Altorjay I, Dotan N, Palatka K, Foldi I (2008). New serological markers for inflammatory bowel disease are associated with earlier age at onset, complicated disease behavior, risk for surgery, and NOD2/CARD15 genotype in a Hungarian IBD cohort.. Am J Gastroenterol.

[49] Lakatos PL, Willheim-Polli C, Folhoffer A (2004). NOD2/CARD15 SNP8, 12 and 13 and other exon4 mutations and primary biliary cirrhosis in Hungarian and Polish patients.. Z Gastroenterol.

[50] Gaj P, Habior A, Mikula M, Otrowski J (2008). Lack of evidence for association of primary sclerosing cholangitis and primary biliary cirrhosis with risk alleles for Crohn's disease in Polish patients.. BMC Med Genet.

[51] Albillos A, de-la-Hera A, Alvarez-Mon M (2004). Serum lipopolysaccharide-binding protein prediction of severe bacterial infection in cirrhotic patients with ascites.. Lancet.

[52] Appenrodt B, Grünhage F, Gentemann MG, Thyssen L, Sauerbruch T (2010). Nucleotide-binding oligomerization domain containing 2 (NOD2) variants are genetic risk factors for death and spontaneous bacterial peritonitis in liver cirrhosis.. Hepatology.

[53] Altorjay I, Vitalis Zs, Tornai I, Palatka K, Kacska S (2010). Mannose-binding lectin deficiency confers risk for bacterial infections in a large Hungarian cohort of patients with liver cirrhosis.. J Hepatol; June 2?Epub ahead of print?.

[54] Rieder F, Schleder S, Wolf A, Dirmeier A, Strauch U (2010). Association of the novel serologic anti-glycan antibodies anti-laminarin and anti-chitin with complicated Crohn's disease behavior.. Inflamm Bowel Dis;.

